# Effect of metformin on kidney function in patients with type 2 diabetes mellitus and moderate chronic kidney disease

**DOI:** 10.18632/oncotarget.23387

**Published:** 2017-12-17

**Authors:** Wei-Hao Hsu, Pi-Jung Hsiao, Pi-Chen Lin, Szu-Chia Chen, Mei-Yueh Lee, Shyi-Jang Shin

**Affiliations:** ^1^ Division of Endocrinology and Metabolism, Department of Internal Medicine, Kaohsiung Medical University Hospital, Kaohsiung Medical University, Kaohsiung, Taiwan; ^2^ Department of Internal Medicine, Kaohsiung Municipal Hsiao-Kang Hospital, Kaohsiung Medical University, Kaohsiung, Taiwan; ^3^ Faculty of Medicine, College of Medicine, Kaohsiung Medical University, Kaohsiung, Taiwan; ^4^ Graduate Institute of Clinical Medicine, College of Medicine, Kaohsiung Medical University, Kaohsiung, Taiwan; ^5^ Division of Nephrology, Department of Internal Medicine, Kaohsiung Medical University Hospital, Kaohsiung Medical University, Kaohsiung, Taiwan; ^6^ Center for Lipid and Glycomedicine Research, Kaohsiung Medical University, Kaohsiung, Taiwan

**Keywords:** metformin, diabetes mellitus, chronic kidney disease, renal function decline, estimated glomerular filtration rate

## Abstract

**Background:**

Impaired renal function can lead to the accumulation of metformin, and elevated concentrations of metformin have been associated with lactic acidosis. The aim of this study was to evaluate the effect of continuous metformin treatment in patients with type 2 diabetes mellitus (DM) and moderate chronic kidney disease (CKD) (estimated glomerular filtration rate (eGFR) 30–0 ml/min/1.73 m^2^) on renal function.

**Methods:**

A total of the 616 patients were enrolled from the research database of Kaohsiung Medical University Hospital from January 1 to 2009 and December 31, 2013. The patients were divided into two groups: those who continued metformin treatment (continuation group; *n* = 484), and those who discontinued metformin treatment for at least 100 days (interruption group; *n* = 132).

**Results:**

The slope of eGFR in the metformin interruption group was statistically lower than that in the metformin continuation group (0.75 ± 0.76 vs. –1.32 ± 0.24 mL/min/1.73 m^2^/year, *p* = 0.0007). After adjusting for baseline covariates in the multivariate linear regression analysis, the continuation of metformin (unstandardized coefficient β, –2.072; 95% confidence interval, –3.268– –0.876) was a risk factor for the patients with DM and moderate CKD.

**Conclusions:**

Metformin may have an adverse effect on renal function in patients with type 2 DM and moderate CKD.

## INTRODUCTION

Metformin is an oral hypoglycemic agent of the biguanide class that lowers blood glucose level mainly by decreasing hepatic glucose production and improving insulin sensitivity of the peripheral tissues by increasing peripheral glucose uptake and utilization [1]. Metformin was approved by the U.S. Food and Drug Administration (FDA) In 1994, and it is generally recommended as the first-line pharmacological agent in management guidelines for patients with type 2 diabetes mellitus (DM) because of its low cost, safety, and association with a reduction in the risk of cardiovascular events [2].

The use of metformin is contraindicated in men and women with serum creatinine concentrations of 1.5 mg/dL or higher and 1.4 mg/dL or higher, respectively, due to the risk of the life-threatening complication, lactic acidosis. Risk factors including severe dehydration (i.e., reduced tissue perfusion), congestive heart failure, sepsis, shock, hypoxia, hepatic impairment, advanced age, and excessive alcohol intake may also increase the risk of metformin-associated lactic acidosis [1, 3, 4]. Metformin is primarily excreted unchanged by the kidney, and renal impairment may cause the accumulation of metformin leading to an elevated metformin concentration, and this has been proposed to lead to lactic acidosis [5]. The overall incidence of lactic acidosis in metformin users ranges from approximately 3 per 100,000 person-years to 10 per 100,000 person-years, which is similar to the background rate in patients with type 2 DM [6].

In April 2016, the FDA revised their warning with regards to metformin use in patients with impaired kidney function, defining the renal impairment according to estimated glomerular filtration rate (eGFR). The revised guidelines stated that the use of metformin is only absolutely contraindicated in patients with severe chronic kidney disease (CKD) (eGFR < 30 ml/min/1.73 m^2^). Therefore, patients with moderate CKD (eGFR 30–59 ml/min/1.73 m^2^) are eligible to receive metformin. A previous study reported that the use of metformin in patients with type 2 DM and advanced CKD was associated with a significantly increased risk of all-cause mortality compared with non-users [7]. This raises the concern that a decline in renal function in patients with moderate CKD receiving metformin may increase the risk of toxicity to metformin. The purpose of this study was to evaluate the effect of continuous metformin treatment on renal function in patients with type 2 DM and moderate CKD (eGFR 30–60 ml/min/1.73 m^2^).

## RESULTS

We identified 23,297 patients who were diagnosed with type 2 DM in the Kaohsiung Medical University Hospital (KMUH) research database between January 1, 2009 and December 31, 2013, of whom 616 met our inclusion and exclusion criteria. One hundred and thirty-two patients were classified to the metformin interruption group, and 484 to the metformin continuation group. Several differences were noted in baseline characteristics between the two groups (Table 1). The patients in the metformin interruption group were younger, had a higher level of glycated hemoglobin (HbA1c), increased use of anti-diabetic drugs including α-glucosidase inhibitors, dipeptidyl peptidase-4 (DDP-4) inhibitors and insulin, and increased use of statins compared to those in the metformin continuation group.

**Table 1:** Baseline clinical characteristics of patients among study groups

Characteristics	Metformin interruption (*n* = 132)	Metformin continuation (*n* = 484)	*P*-value
Age (year)	65.9 ± 9.7	68.3 ± 10.2	0.0190^*^
Male gender (%)	63 (47.7)	212 (43.8)	0.4213
Comorbidities of baseline			
Hypertension (%)	53 (40.2)	171 (35.3)	0.3074
Ischemic heart disease (%)	9 (6.8)	15 (3.1)	0.0503
Heart failure (%)	2 (1.5)	6 (1.2)	0.6824^a^
Categories of UACR			0.1298
UACR <30 mg/dL (%)	67 (50.8)	278 (57.4)	
UACR 30–299 mg/dL (%)	47 (35.6)	129 (26.7)	
UACR ≥300 mg/dL (%)	18 (13.6)	77 (15.9)	
Laboratory parameters			
Fasting glucose (mg/dL)	142.3 ± 49.4	147.4 ± 52.2	0.4087
Triglyceride (mg/dL)	177.5 ± 126.5	156.8 ± 114.8	0.0730
Total cholesterol (mg/dL)	173.9 ± 42.9	176.8 ± 43.0	0.4976
LDL-C (mg/dL)	99.5 ± 34.8	101.1 ± 33.6	0.6475
HDL-C (mg/dL)	38.9 ± 12.6	41.0 ± 11.9	0.0877
HbA1C (%)	8.3 ± 1.8	7.9 ± 2.0	0.0239^*^
Baseline eGFR (mL/min/1.73 m^2^)	48.2 ± 8.2	49.4 ± 7.7	0.0999
Uric acid (mg/dL)	7.2 ± 2.1	7.1 ± 1.8	0.5736
Anti-hypertensive drugs			
ACEI and/or ARB (%)	118 (89.4)	437 (90.3)	0.7602
Calcium channel blocker (%)	95 (72.0)	364 (75.2)	0.4494
β-blocker (%)	65 (49.2)	206 (42.6)	0.1705
Diuretics (%)	81 (61.4)	308 (63.6)	0.6314
Anti-diabetic drugs			
Sulfonylurea (%)	111 (84.1)	388 (80.2)	0.3081
Meglitinide(%)	26 (19.7)	67 (13.8)	0.0959
α-glucosidase inhibitor (%)	38 (28.8)	91 (18.8)	0.0124^*^
Thiazolidinedione (%)	58 (43.9)	206 (42.6)	0.7768
DDP4 inhibitor (%)	75 (56.8)	218 (45.0)	0.0163^*^
Insulin (%)	58 (43.9)	164 (33.9)	0.0329^*^
Lipid-lowering drugs			
Statin (%)	105 (79.6)	336 (69.4)	0.0222^*^
Fibrate (%)	37 (28.0)	101 (20.9)	0.0802
Niacin (%)	2 (1.5)	8 (1.7)	0.9999^a^
Cox-1+Cox-2 inhibitor (%)	38 (28.8)	153 (31.6)	0.5341

Abbreviations. UACR, urinary albumin creatinine ratio; LDL-C, low-density lipoprotein cholesterol; HDL-C, high-density lipoprotein cholesterol; eGFR, estimated glomerular filtration rate; ACEI, angiotensin converting enzyme inhibitor; ARB, angiotensin II receptor blocker; DDP-4, dipeptidyl peptidase-4; Cox, cyclooxygenase.

^*^P < 0.05 compared with the group with metformin continuation. A *p*-value of < 0.05 was considered to be statistically significant.

**Figure 1:**
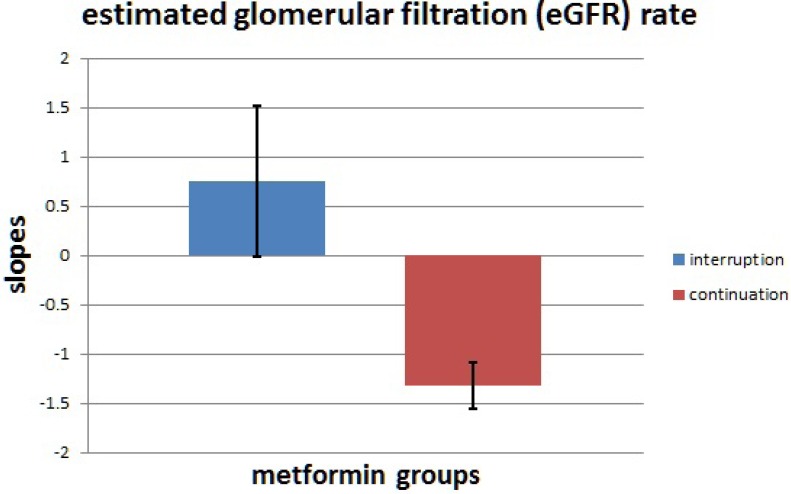
The estimated glomerular filtration rate (eGFR) slopes The eGFR slopes in two groups with metformin interruption versus metformin continuation were 0.75 *±* 0.76 and –1.32 *±* 0.24 mL/min/1.73 m^2^/year, respectively. The eGFR slope was lower in the group with metformin continuation than in the group with metformin interruption (*p* = 0.0007).

The eGFR slopes in the metformin interruption and continuation groups were 0.75 *±* 0.76 and –1.32 *±* 0.24 mL/min/1.73 m^2^/year, respectively (Figure 1), and the eGFR slope was significantly lower in metformin continuation group (*p* = 0.0007). In univariate linear regression analysis (Table 2), the risk of a decline in the eGFR slope was associated with metformin continuation (unstandardized coefficient β, –2.072; 95% confidence interval (CI), –3.268– –0.876), high serum low-density lipoprotein cholesterol (LDL-C) (unstandardized coefficient β, –0.024; 95% CI, –0.039 – –0.010), high HbA1c (unstandardized coefficient β, –0.394; 95% CI, –0.641 – –0.146), low baseline eGFR (unstandardized coefficient β, 1.697; 95% CI, 0.625–2.770), high uric acid level (unstandardized coefficient β, –0.454; 95% CI, –0.766 – –0.142), high urinary albumin creatinine ratio (UACR) (unstandardized coefficient β, –1.339; 95% CI, –1.998 – –0.679), use of angiotensin-converting enzyme inhibitors (ACEIs) and/or angiotensin receptor blockers (ARBs) (unstandardized coefficient β, –3.667; 95% CI, –5.30 – –2.034), and the use of cyclooxygenase-1 (COX-1) or COX-2 inhibitors (unstandardized coefficient β, 1.407; 95% CI, –0.641 – –0.146). In multivariate linear regression analysis (Table 2), the risk of a decline in the eGFR slope was associated with metformin continuation (unstandardized coefficient β, –2.339; 95% CI, –3.632 – –1.047), high serum LDL-C (unstandardized coefficient β, –0.025; 95% CI, –0.041 – –0.008), high HbA1c (unstandardized coefficient β, –0.321; 95% CI, –0.593 – –0.049), low baseline eGFR (unstandardized coefficient β, 2.599; 95% CI, 1.399–3.799), high uric acid level (unstandardized coefficient β, –0.480; 95% CI, –0.777 – –0.184), high UACR (unstandardized coefficient β, –1.133; 95% CI, –1.875 – –0.391), and the use of ACEIs and/or ARBs (unstandardized coefficient β, –3.593; 95% CI, –5.487 – –1.698).

**Table 2:** Determinants of eGFR slope using linear regression analysis

Characteristics	Univariate		Multivariate	
	Unstandardized coefficient β (95% CI)	*p*	Unstandardized coefficient β (95% CI)	*p*
Metformin-continuation vs. Metformin-interruption	–2.072 (–3.268, –0.876)	0.0007^*^	–2.253 (–3.549, –0.957)	0.0007^*^
Age (per 1 year)	–0.0004 (–0.049, 0.048)	0.9858		
Male vs. female	0.611 (–0.384, 1.606)	0.2294		
Hypertension	0.377 (–0.652, 1.406)	0.4732		
Ischemic heart disease	2.367 (–0.186, 4.921)	0.0697	2.481 (–0.174, 5.136)	0.0676
Heart failure	1.376 (–2.999, 5.750)	0.5379		
Laboratory parameters				
Fasting glucose (per 1 mg/dL)	–0.006 (–0.017, 0.006)	0.3383		
Triglyceride (per 1 mg/dL)	0.003 (–0.001, 0.007)	0.1237		
Total cholesterol (per 1 mg/dL)	–0.011 (–0.023, 0.000)	0.0518	0.015 (–0.004, 0.035)	0.0350^*^
LDL-C (per 1 mg/dL)	–0.024 (–0.039, –0.010)	0.0011^*^	–0.039 (–0.064, –0.015)	0.0020^*^
HDL-C (per 1 mg/dL)	0.035 (–0.007, 0.077)	0.1070		
HbA1C (per 1 %)	–0.394 (–0.641, –0.146)	0.0019^*^	–0.333 (–0.606, –0.06)	0.0170^*^
Baseline eGFR (per 1 mL/min/1.73 m^2^)	1.697 (0.625, 2.770)	0.0020^*^	2.604 (1.404, 3.805)	<.0001^*^
Uric acid (per 1 mg/dL)	–0.454 (–0.766, –0.142)	0.0045^*^	–0.482 (–0.779, –0.185)	0.0016^*^
UACR (per 1 mg/dL)	–1.339 (–1.998, –0.679)	<.0001^*^	–1.176 (–1.923, –0.429)	0.0021^*^
Anti-hypertensive drugs				
ACEI and/or ARB	–3.667 (–5.30, –2.034)	<.0001^*^	–3.439 (–5.393, –1.484)	0.0006^*^
Calcium channel blocker	–0.809 (–1.944, 0.326)	0.1627		
β-blocker	–0.473 (–1.470, 0.525)	0.3533		
Diuretics	–0.880 (–1.905, 0.144)	0.0927	0.019 (–1.184, 1.221)	0.9756
Anti-diabetic drugs				
Sulfonylurea	0.685 (–0.577, 1.946)	0.2880		
Meglitinide	0.124 (–1.260, 1.508)	0.8607		
α-glucosidase inhibitor	0.603 (–0.614, 1.819)	0.3318		
Thiazolidinedione	0.247 (–0.754, 1.248)	0.6284		
DDP4 inhibitor	0.770 (–0.221, 1.760)	0.1282		
Insulin	–0.379 (–1.410, 0.653)	0.4722		
Lipid-lowering drugs				
Statin	–0.156 (–1.254, 0.943)	0.7812		
Fibrate	0.537 (–0.650, 1.725)	0.3753		
Niacin	2.135 (–1.782, 6.052)	0.2857		
Cox-1+Cox-2 inhibitor	1.407 (0.342, 2.472)	0.0099^*^	1.21 (0.021, 2.399)	0.0467^*^

Covariates with *p* value < 0.1 in univariate analysis were entered into multivariate linear regression models. A *p*-value of < 0.05 was considered to be statistically significant.

Abbreviation as Table 1.

## DISCUSSION

In this retrospective cohort study, the patients with type 2 DM who received metformin therapy for at least 6 months had a greater decline in eGFR if they continued metformin therapy compared to those who discontinued metformin treatment for at least 100 days. The continuation of metformin therapy was significantly associated with a decline in renal function in the patients with DM and moderate CKD. Other risk factors for a decline in renal function included high serum LDL-C, high HbA1c, low baseline eGFR, high uric acid level, high UACR, and the use of ACEIs and/or ARBs.

Diabetic nephropathy (DN) is a clinical syndrome characterized by persistent albuminuria confirmed on at least two occasions 3–6 months apart, a progressive decline in glomerular filtration rate, and elevated arterial blood pressure [8]. DN is a microvascular complication of type 2 DM. A previous study reported that metformin was beneficial for improving albuminuria compared with glibenclamide in patients with DN [9]. In addition, an animal study showed that metformin suppressed diabetes-induced podocyte loss in DN by preventing oxidative injury [10]. Recently, Samira et. reported no increase in the risk of acute kidney injury in patients receiving metformin compared to those without metformin treatment by baseline eGFR [11]. However, in the present study, continuous metformin therapy was shown to worsen renal function in patients with DM and moderate CKD. A possible mechanism by which metformin increases the concentration of plasma lactate is that it inhibits mitochondrial respiration in tissues including the liver and muscles responsible for lactate removal [4, 12, 13]. Both the liver and kidney are major lactate-consuming organs, accounting for ∼60% and ∼30% of lactate removal, respectively [14, 15]. Most of the lactate removal by the kidneys is through lactate metabolism rather than excretion [15], and impaired renal function may decrease the ability of the kidneys to metabolize an increase in lactate caused by metformin. A previous clinical study demonstrated that metformin users with a renal function of eGFR <60 mL/min/1.73 m^2^ had a higher risk of lactic acidosis or elevated lactate concentrations [16]. Metformin-associated lactic acidosis can cause metabolic acidosis in patients with moderate CKD, and this has been shown to have a deleterious effect on renal function leading to a decline in eGFR and progression of CKD [17–19]. Several factors have been associated with the effect of metabolic acidosis on the decline in renal function, including ammonia-induced complement activation and acidosis-induced increased production of endothelin and aldosterone [19]. Taken together, all of these factors can cause tubule-interstitial injury and mediate a decline in eGFR [19], which may explain the inconsistent findings with regards to the effect of metformin on renal function.

Diabetic nephropathy is associated with oxidative stress caused by a persistent hyperglycemic state and increase in advanced glycation end products [20]. Hyperglycemia has been shown to alter redox equilibrium, which can then induce oxidative stress and cause kidney damage [21]. An animal study showed that the direct anti-oxidation and anti-inflammation effects of tanshinone II, a major diterpenoid derived from Salvia miltiorrhiza, alleviated renal histopathological injuries [22]. This suggests that oxidative stress mediates glucose-induced renal injury, and that alleviating oxidative injury may provide a renal protective effect. Hyperglycemia, inflammation, and cytokines have been reported to cause epigenetic modifications [23–25], including cytosine methylation of DNA (DNA methylation), histone posttranslational modifications and noncoding RNA [26]. Moreover, a review article showed that epigenetic processes are involved in the pathogenesis of DN [20]. Metformin has been shown to participate in DNA methylation by promoting DNA methyltransferase [27]. Hyperglycemia, oxidative stress, inflammation, cytokines, and gene and epigenetic modification may have complex interactions in which metformin may be involved, causing the progression of DN in patients with CKD.

Dyslipidemia has been reported to be a risk factor for adverse renal outcomes in apparently healthy men [28] and in patients with stage 3–5 CKD [29]. The pathophysiological link between dyslipidemia and CKD has been reported to involve accelerated atherosclerosis in the renal microcirculation and deposition of lipoprotein in glomerular structures [30]. Several animal studies have shown that a high total cholesterol level can accelerate the rate of progression of kidney disease, and that a high-cholesterol feeding can lead to macrophage infiltration and foam cell formation in rats [30, 31]. In addition, a low eGFR and high UACR at baseline have been associated with an increased risk of renal events in patients with type 2 DM [32]. Moreover, an elevated baseline HbA1c level has been reported to be a predictive factor for the progression of diabetic kidney disease [33]. In the present study, high serum LDL-C, high HbA1c, low baseline eGFR, and high UACR were associated with a decline in renal function, which is consistent with previous studies.

There are several limitations to this study. First, this was a retrospective study that did not include randomization of the samples, and causation cannot be inferred from the results. Second, this study did not include all confounding factors (e.g. smoking, body weight, duration of DM, liver disease, alcohol abuse). Lastly, ACEIs and ARBs have been shown to reduce neointimal proliferation and vascular inflammation [34]. However, we found that the use of ACEIs or ARBs may have caused a decline in the eGFR slope. We did not evaluate the effects of these anti-hypertensive medications on renal outcomes because this study was not a clinical trial aimed at investigating the effect of medications. We also lacked sufficient data on the cumulative duration of exposure and defined daily dose, and the negative correlation between the use of anti-hypertensive medications and renal outcomes may have been due to selection bias.

In conclusion, continuous metformin treatment in patients with DM and moderate CKD was associated with a worsening in renal function. Metformin is currently indicated for diabetic patients with eGFR > 30 ml/min/1.73 m^2^. However, according to the findings of the present study, metformin should be prescribed with caution for patients with DM and moderate CKD, and renal function should be followed closely in these patients.

## MATERIALS AND METHODS

### Participants

We performed this single-center retrospective cohort study at KMUH in Taiwan. The inclusion criteria were outpatients aged 18 years or older who had been diagnosed with type 2 DM for ≥ 1 year, an eGFR 30–60 ml/min/1.73m^2^, and at least 6 months of metformin treatment. The exclusion criteria were patients with type 1 DM, those who were pregnant, those with chronic glomerulonephritis, kidney transplant recipients, kidney infections, hydronephrosis, calculus of the kidney or ureter, cystitis, and those with bladder or renal cancer. A total of 616 patients were enrolled from the research database of KMUH between January 1, 2009 and December 31, 2013. The patients were divided into two groups according to whether they continuously received metformin treatment (*n* = 484; metformin continuation group) or discontinued metformin treatment for at least 100 days (*n* = 132; metformin interruption group). All patients were followed up for at least one year. The study protocol was approved by the Institutional Review Board of Kaohsiung Medical University Hospital (KMUHIRB-E(I)-20150185).

### Demographic, medical, and laboratory data collection

Demographic and medical data including age, gender, comorbid conditions involving hypertension, ischemic heart disease, heart failure, cerebrovascular disease, peripheral arterial occlusive disease, and liver cirrhosis, and medications including anti-hypertensive agents, anti-diabetic agents, lipid-lowering agents, non-steroidal anti-inflammatory drugs, and selective COX-2 inhibitors were obtained from the research database of KMUH. Baseline laboratory data including blood fasting glucose, serum triglycerides, serum total cholesterol, serum LDL-C, serum high-density lipoprotein cholesterol, HbA1c, eGFR, serum creatinine, serum uric acid, and UACR were also collected. eGFR was calculated using the four-variable equation in the Modification of Diet in Renal Disease study.

### The eGFR slope determinants

eGFR was recorded in the metformin continuation group until the point of the metformin interruption or the end of the study period. In the metformin interruption group, eGFR was measured from at least 100 days after discontinuing metformin until the point of re-starting metformin treatment or December 31, 2013. The rate of renal function decline was determined according to the eGFR slope, and defined as the regression coefficient between eGFR and time in units of mL/min/1.73 m^2^/year, representing the change in the eGFR over time. At least four eGFR values were required to calculate the eGFR slope using regression coefficients between eGFR and time. A faster decline in renal function was indicated by a higher negative eGFR slope.

### Statistical analysis

All statistical analyses were conducted using SAS statistical software version 9.2 (SAS Institute, Cary, NC, USA) for Windows. Data were expressed as mean ± standard deviation. Between group differences were determined using the chi-square test for categorical variables and the independent *t*-test for continuous variables. A mixed-effect model analysis was used to evaluate the eGFR slopes between groups. A linear regression analysis was performed to determine the eGFR slopes. Covariates were entered into multivariate linear regression models if their *p* value was < 0.1 in univariate analysis. A *p*-value of < 0.05 was considered to be statistically significant.

## Abbreviations

DM: diabetes mellitus
CKD: chronic kidney disease
eGFR: estimated glomerular filtration rate
KMUH: Kaohsiung Medical University Hospital
FDA: Food and Drug Administration
HbA1c: glycated hemoglobin
DDP-4: dipeptidyl peptidase-4
LDL-C: low-density lipoprotein cholesterol
UACR: urinary albumin creatinine ratio
ACEI: angiotensin-converting enzyme inhibitor
ARB: angiotensin receptor blocker
COX-1: cyclooxygenase-1
DN: diabetic nephropathy
HDL-C: high-density lipoprotein cholesterol
